# Supplementary material for: Non-Specific Effects of Prepartum Vaccination on Uterine Health and Fertility: A Retrospective Study on Periparturient Dairy Cows
Source: Animals (Basel). 2025 Sep 3;15(17):2589. doi: 10.3390/ani15172589 (PMC12427402; doi:10.3390/ani15172589)
Supplement: Supplementary file 1 [file animals-15-02589-s001.zip › File S3.docx]

**Table S2. Results of multivariable analysis of the association between time of prepartum vaccination and uterine health and fertility in non-live vaccinated cows.**

| **Postpartum time interval** | **Day 1-21** | | | **Day 22-56** | | | **Appr. > Day 100** | | |
| --- | --- | --- | --- | --- | --- | --- | --- | --- | --- |
|  | **RMC^a^** | | | **Endometritis^b^** | | | **NRR56^c^** | | |
|  | **OR^d^** | **(95% CI^e^)** | **p-value^f^** | **OR^d^** | **(95% CI^e^)** | **p-value^f^** | **OR^d^** | **(95% CI^e^)** | **p-value^f^** |
| **Time of vaccination^g^** |  |  |  |  |  |  |  |  |  |
| LATE / EARLY | 0.69 | 0.33, 1.47 | 0.341 | 0.37 | 0.10, 1.35 | 0.133 | 1.42 | 1.12, 1.79 | **0.004** |
| **Multiples** | 6.88 | 5.51, 8.60 | **<0.001** | 1.28 | 1.00, 1.65 | 0.050 | 0.76 | 0.60, 0.95 | 0.016 |
| **Dystocia** | 1.24 | 1.07, 1.44 | 0.005 | 1.50 | 1.29, 1.74 | **<0.001** |  |  |  |
| **Stillbirth** | 4.00 | 3.18, 5.03 | **<0.001** | 1.19 | 0.92, 1.54 | 0.178 |  |  |  |
| **Calf gender** | 1.24 | 1.10, 1.39 | **<0.001** |  |  |  |  |  |  |
| **Parity** | 3.19 | 2.33, 4.35 | **<0.001** |  |  |  | 1.12 | 0.90, 1.39 | 0.311 |
| **Access to pasture^i^** | 1.96 | 0.97, 3.93 | 0.059 | 1.63 | 0.45, 5.88 | 0.454 |  |  |  |
| **Hygiene^j^** | 0.93 | 0.59, 1.45 | 0.736 |  |  |  |  |  |  |
| **Dry period^k^** | 1.05 | 0.95, 1.17 | 0.352 |  |  |  |  |  |  |
| **ECM FTD^l^** | 0.95 | 0.94, 0.95 | **<0.001** |  |  |  | 0.99 | 0.99, 1.00 | 0.011 |
| **Risk of ketosis^m^** |  |  |  | 1.13 | 0.99, 1.30 | 0.071 |  |  |  |
| **RMC^a^** |  |  |  | 3.09 | 2.68, 3.56 | **<0.001** |  |  |  |
| **Calving season^n^** |  |  |  |  |  |  |  |  |  |
| spring / autumn | 1.23 | 0.98, 1.55 | 0.096 |  |  |  |  |  |  |
| summer / autumn | 1.20 | 0.97, 1.49 | 0.122 |  |  |  |  |  |  |
| summer / spring | 0.98 | 0.79, 1.22 | 0.994 |  |  |  |  |  |  |
| winter / autumn | 1.12 | 0.90, 1.40 | 0.549 |  |  |  |  |  |  |
| winter / spring | 0.91 | 0.73, 1.14 | 0.715 |  |  |  |  |  |  |
| winter / summer | 0.93 | 0.75, 1.15 | 0.831 |  |  |  |  |  |  |
| ^a^Aggregation of Retained placenta and Metritis within day 1-21 postpartum; ^b^Endometritis within day 22-56 postpartum; ^c^Non-Return-Rate refers to day 56 after first service; ^d^Odds Ratio for pairwise contrasts; ^e^Confidence Interval; ^f^p-values are marked bold, if below the adapted significance threshold, of 0.005; ^g^vaccination between 2.5 and 4 weeks before expected calving date; ^h^vaccination between 6 and 8 weeks before expected calving date; ^i^access to pasture during dry period; ^j^Hygiene score between 1 and 4, documented on the date of on-site survey; ^k^Length of the dry period; ^l^Energy corrected milk yield on the first day of milk testing; ^m^Ketotic risk was assumed, if the fat-protein-ratio exceeds 1.4 and lower limits of protein content (Emin) are undercut or upper limits of fat content (Fmax) are passed on the first day of milk testing. Emin = (4,11 -0,023 kg milk/day) (1 - 0,35/3,51). Fmax = (5,06 -0,033 kg milk/day) (1 + 0,68/4,20); ^n^spring (March-May), summer (June-August), autumn (September-November), winter (December-February).  The variables herd and calving year were applied as random effects. Empty fields arise because the variable was either not significant in the corresponding univariable analysis or was eliminated by manual backward selection. The vertical wiggly line separates NRR56 from the other response variables due to an inverse effect as compared to RMC and Endometritis: while higher RMC and Endometritis rates are undesirable, higher NNR56 rates represent better fertility. | | | | | | | | | |
